# Investigation of traffic behavior in driving accidents in Azarshahr city drivers

**Published:** 2019-08

**Authors:** Mohammad Dashti, M Vakili, M Dahim, M Farahbakhsh, F Torabi, H Bagerpour, Moharami E, Jaffari A

**Affiliations:** ^*a*^Occupational Health Engineering team, Local Health Center of Azarshahr, Tabriz University of Medical Sciences, Tabriz, Iran.; ^*b*^School Health Services, Local Health Center of Azarshahr, Tabriz University of Medical Sciences, Tabriz, Iran.

## Abstract

**Background:**

Traffic accidents and losses caused by it are one of the current challenges of human societies that endanger the health of humans and impose a lot of economic costs on the economy of countries. One of the factors in driving accidents is human factors or driving behavior pattern. The pattern of driving behavior, like other human behavior, is influenced by a set of conscious and unconscious factors that are considered as the overall traffic behavior. The aim of this study was to investigate traffic behavior in driving accidents with Manchester driving behavior questionnaire among personal car drivers in Azarshahr city.

**Methods:**

This study is a research review. It is descriptive-analytic and the necessary information in this design through The Manchester driving behavior questionnaire was collected between 260 drivers. The research environment is the level of Azarshahr city and all the drivers. The data were analyzed by SPSS software version 16.To evaluate the normal distribution of quantitative variables, Kolmogorov-Smirnov test was used and considering that the distribution of variables was not normal for analyzing the difference, Mann-Whitney Test and Spearman test were used for correlation analysis.

**Results:**

In this study, the Manchester Driving behavior questionnaire, four factors of slippage, intentional violations, errors, and intentional driving violations were clearly differentiated among personal car drivers. In the first factor, the mean score of 1.5, second factor (intentional violations) of 1.85, Third factor (errors) 1.34, the fourth factor (unintentional violation) 1.51.In addition, there was a significant difference in demographic variables between men and women in terms of age, driving history, daily driving hours, and accident number (P <0.001).

**Conclusions:**

The findings of this study confirmed that the results of intentional driving violations of the highest score and driving slippage are the second rank of driving behaviors among personal vehicles ' drivers in Azarshahr city.

**Keywords:**

Manchester questionnaire, Driving behavior, Personal vehicle drivers

